# Supplementary material for: Structural and functional characterization of a DARPin which inhibits Ras nucleotide exchange
Source: Nat Commun. 2017 Jul 14;8:16111. doi: 10.1038/ncomms16111 (PMC5519984; doi:10.1038/ncomms16111)
Supplement: Supplementary Information [file ncomms16111-s1.pdf]

File name: Supplementary Information
Description: Supplementary figures and supplementary tables.

File name: Peer review file
Description:

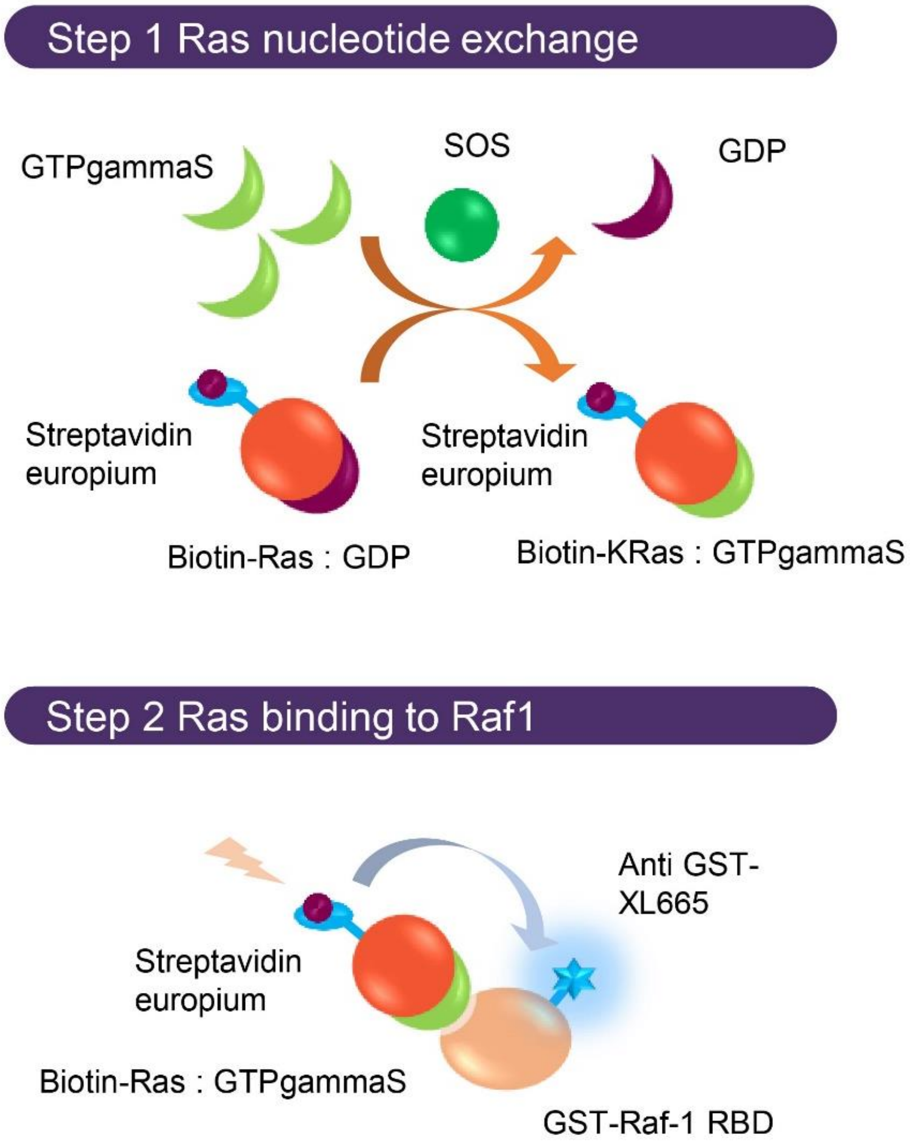

Supplementary Figure 1. Schematic of Ras biochemical coupled assay. The two-step Ras biochemical coupled assay used to identify inhibitory DARPins in this work is described in the schematic diagram. To assay mechanistically for DARPins inhibiting nucleotide exchange or Ras/Raf binding, DARPins were incubated with Biotin-Ras:GDP prior to Step 1, which comprised of Sos-mediated nucleotide exchange, as initiated by the simultaneous addition of Sos, GTPγS and GST-Raf-1/XL665 to biotinylated Ras:GDP. To assay for DARPins which inhibited only the Ras:Raf-1 interaction, DARPins were added prior to step 2, in which GTPγS:Ras is detected upon binding to GST-Raf1-RBD due to the proximity of the streptavidin-europium and anti-GST-XL665.

DARPin	Repeat1																																Coupled assay	Ras/Raf assay	MANT assay	Mechanism
K17	H	D	T	F	G	F	T	S	L	H	L	A	A	L	Y	G	H	L	E	I	V	E	V	L	L	K	D	G	A	D	V	N	A	+	-	+	NE
K19	S	D	R	W	G	W	T	P	L	H	L	A	A	W	W	G	H	L	E	I	V	E	V	L	L	K	R	G	A	D	V	S	A	+	-	+	NE
K27	H	D	T	F	G	F	T	P	L	H	L	A	A	L	Y	G	H	L	E	I	V	E	V	L	L	K	N	G	A	D	V	N	A	+	-	+	NE
K27 Null3	H	D	T	F	G	F	T	P	L	H	L	A	A	L	Y	G	H	L	E	I	V	E	V	L	L	K	N	G	A	D	V	N	A	-	-	-	-
K26	T	D	I	R	G	S	T	P	L	H	L	A	A	L	W	G	H	L	E	I	V	E	V	L	L	K	N	G	A	D	V	N	A	+	+	-	RR
K28	F	D	H	H	G	W	T	P	L	H	L	A	A	Q	Q	G	H	L	E	I	V	E	V	L	L	K	Y	G	A	D	V	N	A	+	+	-	RR
K55	N	D	S	A	G	H	T	P	L	H	L	A	A	K	R	G	H	L	E	I	V	E	V	L	L	K	H	G	A	D	V	N	A	+	+	-	RR
DARPin	Repeat2																																			
K17	D	D	S	Y	G	R	T	P	Q	H	L	A	A	M	R	G	H	L	E	I	V	E	A	L	L	K	Y	G	A	D	V	N	A				
K19	A	D	L	H	G	Q	S	P	L	H	L	A	A	M	V	G	H	L	E	I	V	E	V	L	L	K	Y	G	A	D	V	N	A				
K27	D	D	S	Y	G	R	T	P	L	H	L	A	A	M	R	G	H	L	E	I	V	E	V	L	L	K	Y	G	A	D	V	N	A				
K27 Null3	D	D	S	Y	G	A	T	P	L	H	L	A	A	M	R	G	H	L	E	I	V	E	V	L	L	K	Y	G	A	D	V	N	A				
K26	N	D	R	M	G	R	T	P	L	H	L	A	A	Y	H	G	H	L	E	I	V	E	V	L	L	K	Y	G	A	D	V	N	A				
K28	D	D	L	F	G	Y	T	P	L	H	L	A	A	W	K	G	H	L	E	I	V	E	V	L	L	K	Y	G	A	D	V	N	A				
K55	M	D	N	T	G	F	T	P	L	H	L	A	A	L	R	G	H	L	E	I	V	E	V	L	L	K	N	G	A	D	V	N	A				
DARPin	Repeat3																																			
K17	A	D	E	E	G	R	T	P	L	H	L	A	A	K	R	G	H	L	E	I	V	E	V	L	L	K	N	G	A	D	V	N	A				
K19	K	D	T	M	G	A	T	P	L	H	L	A	A	R	S	G	H	L	E	I	V	E	E	L	L	K	N	G	A	D	M	N	A				
K27	A	D	E	E	G	R	T	P	L	H	L	A	A	K	R	G	H	L	E	I	V	E	V	L	L	K	N	G	A	D	V	N	A				
K27 Null3	A	D	E	E	G	A	T	P	L	H	L	A	A	K	A	G	H	L	E	I	V	E	V	L	L	K	N	G	A	D	V	N	A				
K26	V	D	L	M	G	R	T	P	L	H	L	A	A	M	K	G	H	L	E	I	V	E	V	L	L	K	N	G	A	D	V	N	A				
K28	M	D	H	H	G	H	T	P	L	H	L	A	A	Q	M	G	H	L	E	I	V	E	V	L	L	K	Y	G	A	D	V	N	A				
K55	Q	D	R	T	G	R	T	P	L	H	L	A	A	K	L	G	H	L	E	I	V	E	V	L	L	K	N	G	A	D	V	N	A				

Supplementary Figure 2. Sequences of Ras-inhibitory DARPins identified in biochemical assays. The amino acid sequences of the DARPin repeat domains are shown. Residues highlighted in blue are the residues randomised in the original DARPin library and which form the antigen-binding interface and residues highlighted in pink were mutated to alanine to create the non-binding variant K27 Null3. N-Cap and C-Cap regions are not shown as the DARPins showed no sequence variability in those regions. To the right is a summary of each DARPin's activity profile (+ active; - inactive) in the three assays used for the initial screening and an assignment of each according to their Ras inhibition mechanism (NE = nucleotide exchange inhibitor; RR = Ras/Raf inhibitor).

a

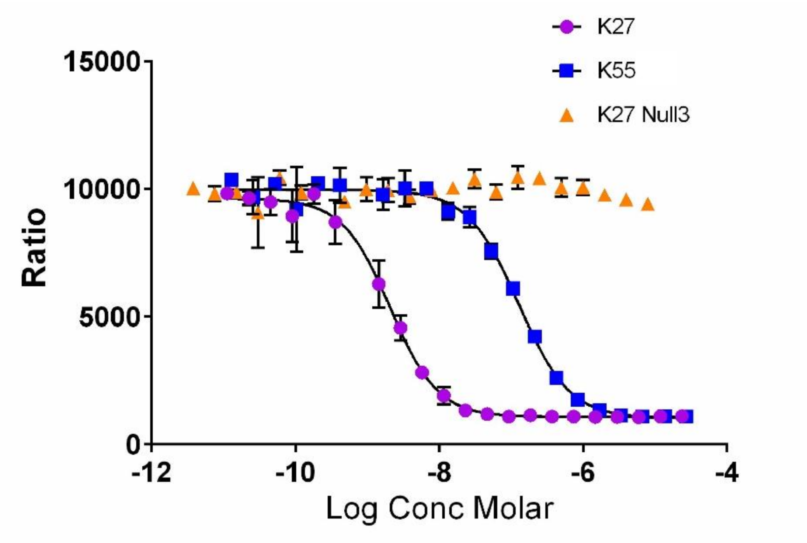

b

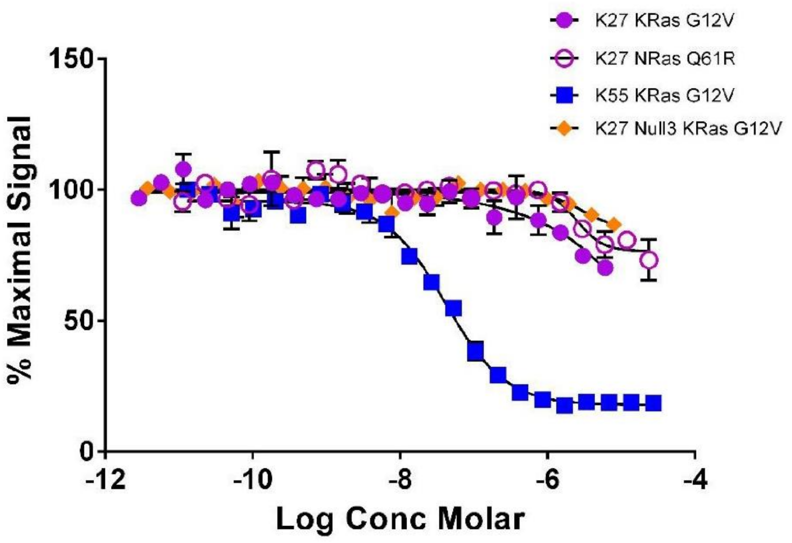

Supplementary Figure 3. Full dose response data for Ras inhibitory DARPins in the Ras biochemical coupled assay and Ras/Raf inhibition assay. (a) Ras biochemical coupled assay for inhibition of nucleotide exchange or Ras/Raf interaction by DARPins K27, K55or K27 Null 3. K-Ras G12V loaded with GDP was incubated for 15 min with dilution series of DARPins K27, K55 or K27 Null 3. Sos and GTPγS were added to allow nucleotide exchange, followed by Raf. The Ras GTPγS/Raf complex was quantitated by the FRET signal. (b) Ras/Raf inhibition assay testing DARPins K27, K55 and K27 Null3. The FRET signal was measured for the interaction between Raf and K-Ras G12V, or N-Ras Q61R loaded with GTPγS and inhibition of the signal monitored at varying concentrations of DARPins K27, K55 and Null 3. For each curve, the FRET signal was normalized by dividing by the signal in the absence of addition of DARPin and multiplying by 100. Each Ras protein loaded with GTPγS contained lower but significant amounts of the GDP form. Error bars represent the mean ± s.d. (n=3).

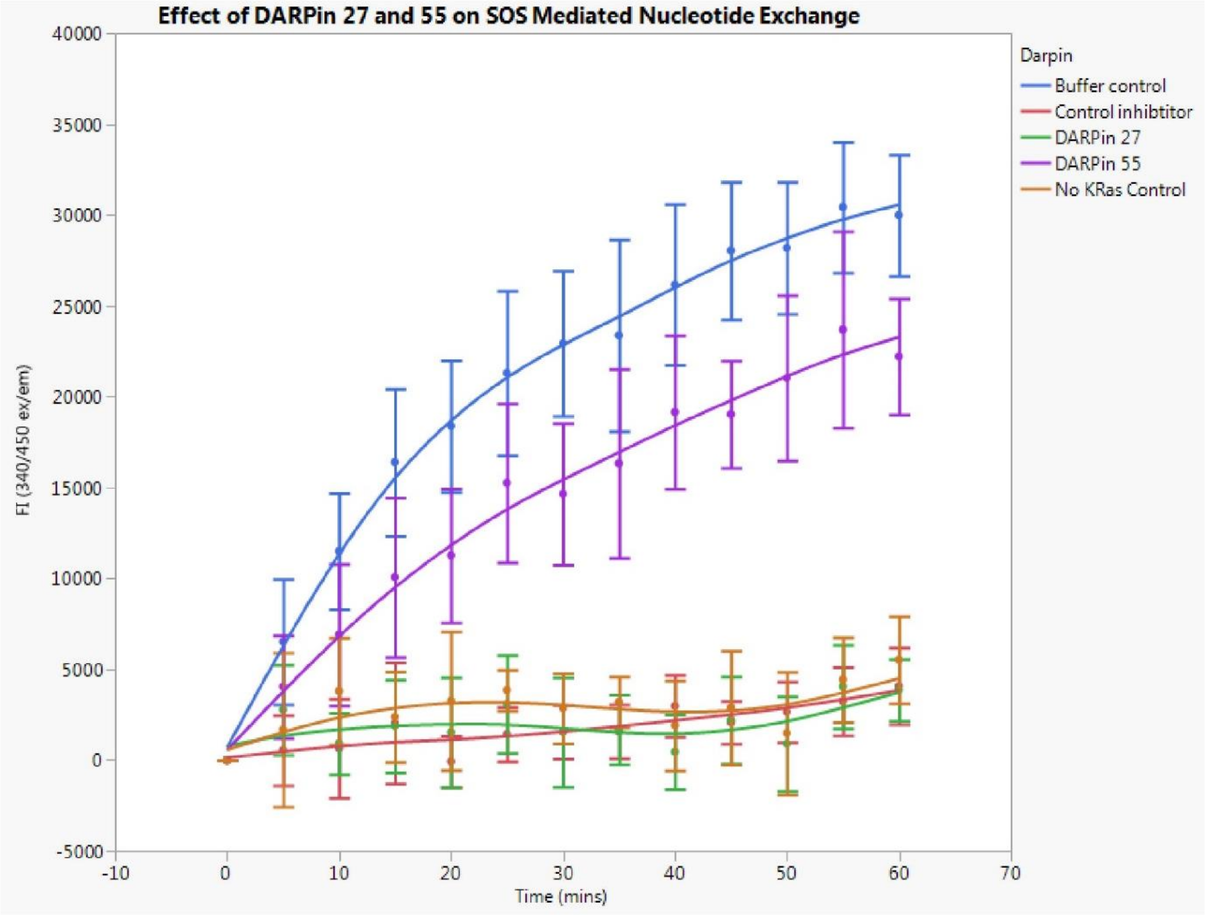

Supplementary Figure 4. DARPin K27 inhibits SOS-mediated nucleotide exchange of Ras G12V in MANT assay. SOS-mediated exchange of GDP with dMANT-GDP (2'-Deoxy-3'-O-(N'-methylanthraniloyl)guanosine-5'-O-diphosphate) on Ras G12V was studied over time in the presence or absence of 10 μM concentrations of inhibitory DARPins K27 or K55. The increase in fluorescence of dMANT-GDP upon binding to Ras G12V as a result of nucleotide exchange was detected by measuring light emission at a wavelength of 450 nm. Error bars represent the mean ± s.d. (n=3).

Supplementary Figure 5. Unprocessed scans of western blots

Figure 5c

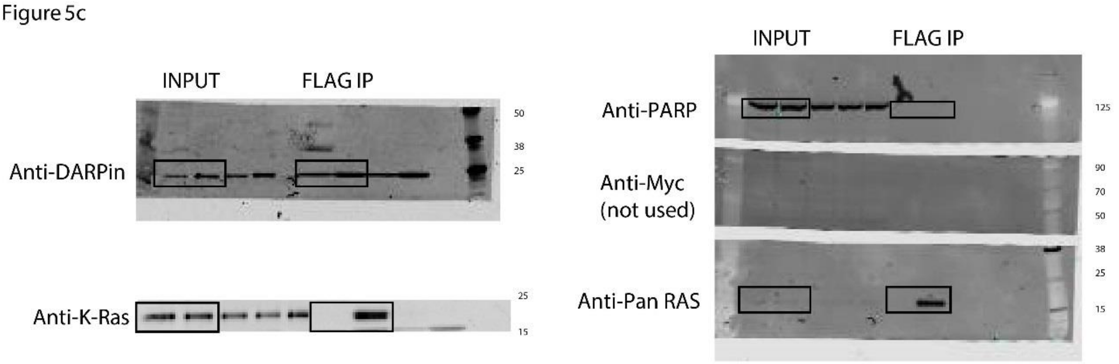

Figure 7a

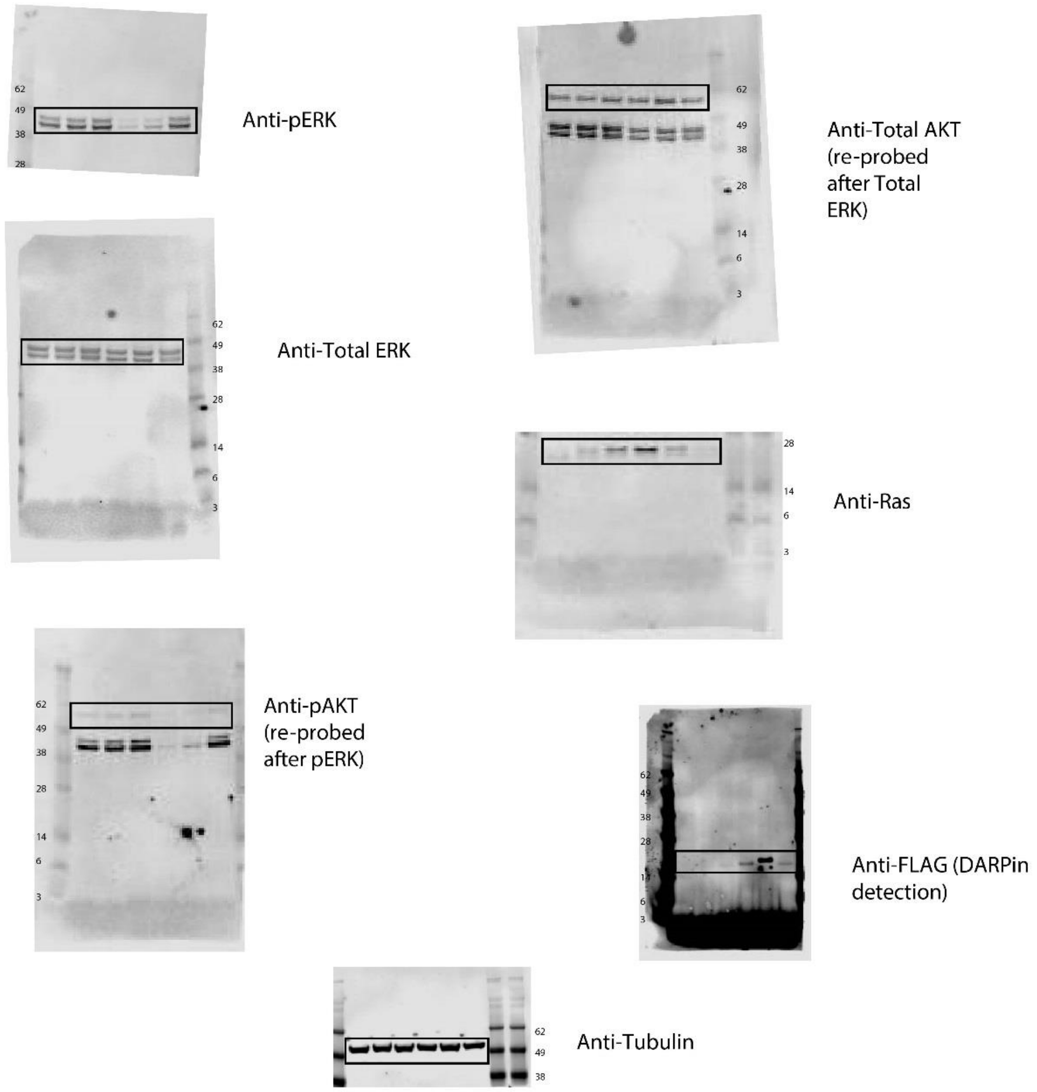

Supplementary Figure 6. Stereo views of part of the electron density maps for both structures

K27/K-Ras G12V structure:

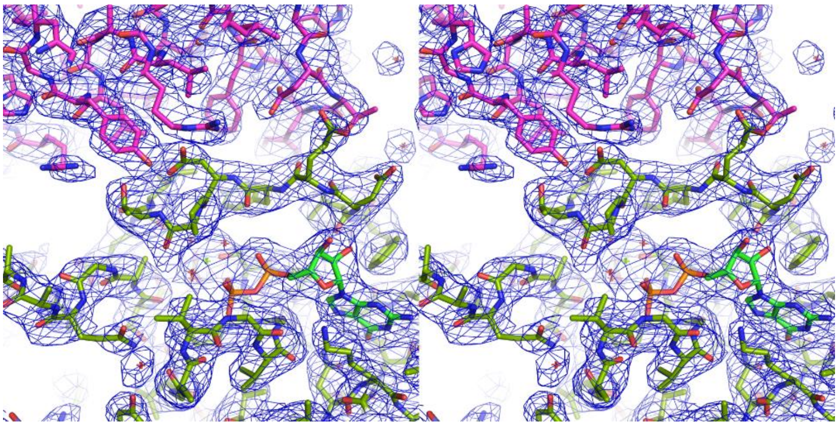

K55/ K-Ras G12V structure:

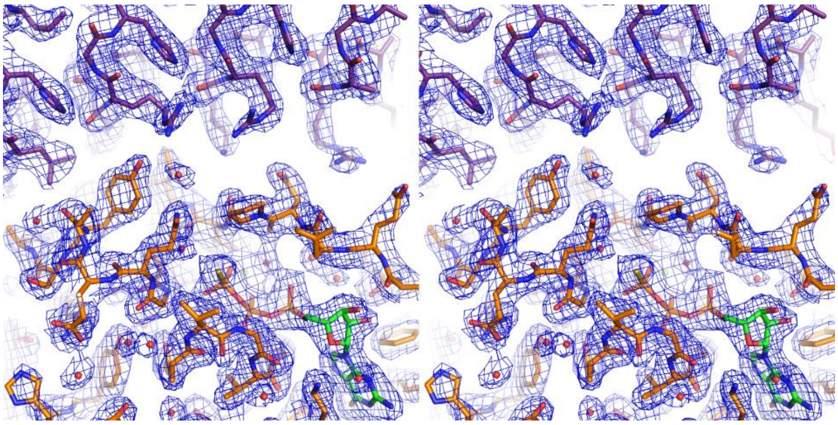

Supplementary Table 1. Kinetic Parameters for DARPins K27 and K55 to Ras isoforms and mutants

DARPin	Isoform	Mutant	Nucleotide Loaded	k_{on} $M^{-1}s^{-1}$	k_{diss} s^{-1}	K_d nM
K27	K-Ras	Wild type	GDP	1.4×10^{5}	5.4×10^{-4}	3.9
K27	K-Ras	G12V	GDP	2.3×10^{5}	6.1×10^{-4}	2.7
K27	K-Ras	G12C	GDP	1.7×10^{5}	5.2×10^{-4}	3.0
K27	K-Ras	G12D	GDP	1.3×10^{5}	4.8×10^{-4}	2.8
K27	N-Ras	G12D	GDP	1.9×10^{5}	5.0×10^{-4}	2.6
K27	N-Ras	Wild type	GDP	2.1×10^{5}	6.2×10^{-4}	3.1
K55	K-Ras	G12V	GTPγS	1.5×10^{5}	2.4×10^{-2}	160
K55	K-Ras	Wild type	GTPγS	1.5×10^{5}	2.5×10^{-2}	167

Supplementary Table 2. Ras biochemical data for DARPins K27 and K55 against Ras isoforms and mutants

DARPin	**K-Ras G12V IC_{50}, nM**	**K-Ras WT IC_{50}, nM**	**K-Ras G12C IC_{50}, nM**	**K-Ras G12D IC_{50}, nM**	**N-Ras WT IC_{50}, nM**	**N-Ras G12D IC_{50}, nM**	**N-Ras Q61R IC_{50}, nM**	**N-Ras Q61K IC_{50}, nM**
Ras Biochemical Coupled Assay:								
K27	2.2	2.4	4.0	1.2	24	1.5	ND	ND
K55	90	67	59	56	94	39	ND	ND
Ras/Raf Assay:								
K27	Incomplete *	Incomplete *	ND	ND	ND	ND	Incomplete *	Incomplete *
K55	64	120	ND	ND	ND	ND	145	81

* Inhibition was incomplete at the maximum K27 concentration of 23.6 μM

ND – not determined

Supplementary Table 3. Ras interaction summary and comparison for DARPin K55, scFv6 and RAF.

Ras Residue	DARPin K55 Interaction	scFv6 Interaction	RAF Interaction
25 Gln	H-bond	H-bond	H-bond
27 His			H-bond
31 Glu	charged		charged
33 Asp		H-bond	
34 Pro	H-bond (mc)	H-bond (mc)	
35 Thr		H-bond	
37 Glu	charged		charged
38 Asp	H-bond	H-bond	charged
39 Ser	H-bond		H-bond
41 Arg			H-bond
54 Asp	charged		
61 Gln	W mediated	W mediated	
64 Tyr	H-bond	H-bond	
70 Gln	H-bond		

H-bond (mc) – main chain hydrogen bond

W mediated – water mediated

Supplementary Table 4. Sequences of genes expressed during this study.

Construct name	DNA sequence
6His-Avi-TEV-NRas_1-172_WT	ATGGGGCATCATCATCACCATCATGGTGGTGGCGGTCTGAATGATATTTTTGAAGCACAGAAAATCGAGTGGCA<br>CGAAGAAAATCTGTATTTTCAGGGTAGCGGTAGCGGATCCCATATGACCGAATATAAACTGGTTGTTGTTGGTG<br>CCGGTGGTGTTGGTAAAAGCGCACTGACCATTCAGCTGATTCAGAATCATTTTGTGGATGAGTATGATCCGACC<br>ATCGAAGATAGTTATCGTAAACAGGTTGTGATTGATGGTGAAACCTGTCTGCTGGATATTCTGGATACCGCAGG<br>TCAAGAGGAATATAGCGCAATGCGTGATCAGTATATGCGTACCGGTGAAGGTTTTCTGTGTGTTTTTGCAATCA<br>ACAACAGCAAATCCTTCGCCGATATTAATCTGTATCGTGAGCAGATTAAACGCGTGAAAGATAGTGATGATGTT<br>CCGATGGTTCTGGTGGGTAATAAATGTGATCTGCCGACCCGTACCGTTGATACCAAACAGGCACATGAACTGGC<br>AAAAAGCTATGGCATTCCGTTTATTGAAACCAGCGCAAAAACCCGTCAGGGTGTTGAAGATGCATTTTATACCC<br>TGGTTCGTGAAATTCGCCAGTACCGTATGAAAAAACTGAACCTCGAGTAATAGAAGCTTACGTAGAC
6His-TEV-Avi-KRas_1-166_G12V	ATGCATCATCATCACCATCATGGCGGTGGCGAAAACCTGTATTTTCAGGGATCCGGTCTGAACGATATTTTTGA<br>GGCACAAAAAATCGAGTGGCACGAACATATGACCGAATATAAACTGGTTGTTGTTGGTGCAGTTGGTGTTGGTA<br>AAAGCGCACTGACCATTCAGCTGATTCAGAATCATTTTGTGGATGAATATGATCCGACCATTGAAGATAGCTAT<br>CGTAAACAGGTGGTGATTGATGGTGAAACCTGTCTGCTGGATATTCTGGATACCGCAGGTCAAGAGGAGTATAG<br>CGCAATGCGCGATCAGTATATGCGTACCGGTGAAGGTTTTCTGTGTGTGTTTGCCATTAATAATACCAAATCCT<br>TTGAAGATATTCATCATTATCGCGAACAAATTAAACGTGTGAAAGATAGCGAAGATGTTCCGATGGTTCTGGTT<br>GGTAATAAATGTGATCTGCCGAGCCGTACCGTTGATACCAAACAGGCACAGGATCTGGCTCGTAGCTATGGTAT<br>TCCGTTTATTGAAACCAGCGCAAAAACCCGTCAGGGTGTGGATGATGCATTTTATACCCTGGTGCGCGAAATTC<br>GCAAACATTAATAGAAGCTTACGTAGAC
KRas_1-166_-Avi-6His_WT	ATGACCGAATATAAACTGGTTGTTGTTGGTGCCGGTGGTGTTGGTAAAAGCGCACTGACCATTCAGCTGATTCA<br>GAATCATTTTGTGGATGAGTATGATCCGACCATCGAAGATAGTTATCGTAAACAGGTTGTGATTGATGGTGAAA<br>CCTGTCTGCTGGATATTCTGGATACCGCAGGTCAAGAGGAATATAGCGCAATGCGTGATCAGTATATGCGTACC<br>GGTGAAGGTTTTCTGTGTGTTTTTGCAATCAACAACACCAAATCCTTCGAAGATATCCATCATTATCGCGAGCA<br>GATTAAACGTGTGAAAGATAGCGAAGATGTTCCGATGGTTCTGGTTGGTAATAAATGTGATCTGCCGAGCCGTA<br>CCGTTGATACCAAACAGGCACAGGATCTGGCACGTAGCTATGGTATTCCGTTTATTGAAACCAGCGCAAAAACC<br>CGTCAGGGTGTTGATGATGCATTTTATACCCTGGTTCGTGAAATCCGCAAACATCTCGAGGGTAGCGGTAGCGG<br>TTCAGGTCTGAATGATATTTTTGAAGCCCAGAAAATCGAATGGCATGAAGGTGGTGGTCATCATCATCACCATC<br>AT
6His-TEV-HRas_1-166_WT	ATGCATCATCATCACCATCATGGCGGTGGCGAAAACCTGTATTTTCAGGGATCCCATATGACCGAATATAAACT<br>GGTTGTTGTTGGTGCAGGTGGTGTTGGTAAAAGCGCACTGACCATTCAGCTGATTCAGAATCATTTTGTGGATG<br>AATATGATCCGACCATTGAAGATAGCTATCGTAAACAGGTGGTGATTGATGGTGAAACCTGTCTGCTGGATATT<br>CTGGATACCGCAGGTCAAGAGGAGTATAGCGCAATGCGCGATCAGTATATGCGTACCGGTGAAGGTTTTCTGTG<br>TGTGTTTGCCATTAATAATACCAAATCCTTTGAAGATATTCATCAGTATCGCGAACAAATTAAACGTGTGAAAG<br>ATTCTGATGATGTTCCGATGGTTCTGGTTGGTAATAAATGTGATCTGGCTGCACGTACCGTTGAAAGCCGTCAG<br>GCACAGGATCTGGCTCGTAGCTATGGTATTCCGTATATTGAAACCAGCGCAAAAACCCGTCAGGGTGTGGAAGA<br>TGCATTTTATACCCTGGTGCGTGAAATTCGCCAGCAT
DARPin K17	GATCTGGGAAAAAAACTGCTGGAAGCCGCGCGTGCCGGGCAGGACGATGAGGTCCGTATTCTTATGGCGAACGG<br>TGCGGATGTTAACGCACACGATACGTTCGGTTTCACGTCGCTGCATCTGGCAGCGCTGTACGGTCACCTCGAAA<br>TTGTGGAAGTGCTGTTGAAGGATGGTGCAGATGTTAACGCGGATGATAGCTACGGTCGCACGCCGCAGCATCTG<br>GCAGCGATGCGCGGTCACCTCGAAATTGTGGAGGCGCTGTTGAAGTACGGTGCAGATGTTAACGCGGCAGATGA<br>GGAGGGTCGCACGCCGCTGCATCTGGCAGCGAAACGCGGTCACCTCGAAATTGTGGAAGTGCTGTTGAAGAATG<br>GTGCAGATGTGAATGCTCAGGATAAGTTTGGCAAAACCGCGTTTGATATCTCCATTGATAATGGCAACGAAGAT<br>TTAGCGGAAATCCTGCAGAAACTG
DARPin K19	GATCTGGGAAAAAAACTGCTGGAAGCCGCGCGTGCCGGGCAGGACGATGAGGTCCGTATTCTTATGGCGAATGG<br>TGCAGATGTTAACGCGAGCGATCGTTGGGGTTGGACGCCGCTGCACCTGGCAGCGTGGTGGGGTCACCTCGAAA<br>TTGTGGAAGTGCTGTTGAAGCGCGGTGCAGATGTTAGCGCGGCAGATCTGCACGGTCAATCGCCGCTGCATCTG<br>GCAGCGATGGTCGGCCACCTCGAAATTGTGGAAGTGCTGTTGAAGTACGGTGCAGATGTTAACGCGAAAGATAC<br>GATGGGTGCAACGCCGCTGCACCTGGCAGCGCGAAGCGGTCACCTCGAAATTGTGGAAGAGCTGTTGAAGAACG<br>GTGCAGATATGAATGCTCAGGATAAGTTTGGCAAAACCACGTTTGATATCTCCACTGATAATGGCAACGAAGAT<br>TTAGCGGAAATCCTGCAGAAACTG
DARPin K26	GATCTGGGAAAAAAACTGCTGGAAGCCGCGCGTGCCGGGCAGGACGATGAGGTCCGTATTCTTATGGCGAACGG<br>TGCAGATGTTAACGCGACGGATATTCGCGGTAGCACGCCGCTGCATCTGGCAGCGCTGTGGGGTCACCTCGAAA<br>TTGTGGAAGTGCTGTTGAAGAATGGTGCAGATGTTAACGCGAACGATCGCATGGGTCGCACGCCGCTGCATCTG<br>GCAGCGTACCACGGTCACCTCGAAATTGTGGAAGTGCTGTTGAAGTACGGTGCAGATGTTAACGCGGTCGATCT<br>GATGGGTCGCACGCCGCTGCATCTGGCAGCGATGAAAGGTCACCTCGAAATTGTGGAAGTGCTGTTGAAGAATG<br>GTGCAGATGTGAATGCTCAGGATAAGTTTGGCAAAACCGCGTTTGATATCTCCATTGATAATGGCAACGAAGAT<br>TTAGCGGAAATCCTGCAGAAACTG
DARPin K27	GATCTGGGAAAAAAACTGCTGGAAGCCGCGCGTGCCGGGCAGGACGATGAGGTCCGTATTCTTATGGCGAACGG<br>TGCAGATGTTAACGCGCACGATACGTTCGGTTTCACGCCGCTGCATCTGGCAGCGCTGTACGGTCACCTCGAAA<br>TTGTGGAAGTGCTGTTGAAGAATGGTGCAGATGTTAACGCGGATGATAGCTACGGTCGCACGCCGCTGCATCTG<br>GCAGCGATGCGCGGTCACCTCGAAATTGTGGAAGTGCTGTTGAAGTACGGTGCAGATGTTAACGCGGCAGATGA<br>GGAGGGTCGCACGCCGCTGCATCTGGCAGCGAAACGCGGTCACCTCGAAATTGTGGAAGTGCTGTTGAAGAATG<br>GTGCAGATGTGAATGCTCAGGATAAGTTTGGCAAAACCGCGTTTGATATCTCCATTGATAATGGCAACGAAGAT<br>TTAGCGGAAATCCTGCAGAAACTG
DARPin K27 null3	GATCTGGGAAAAAAACTGCTGGAAGCCGCGCGTGCCGGGCAGGACGATGAGGTCCGTATTCTTATGGCGAACGG<br>TGCAGATGTTAACGCGCACGATACGTTCGGTTTCACGCCGCTGCATCTGGCAGCGCTGTACGGTCACCTCGAAA<br>TTGTGGAAGTGCTGTTGAAGAATGGTGCAGATGTTAACGCGGATGATAGCTACGGTgcCACGCCGCTGCATCTG<br>GCAGCGATGCGCGGTCACCTCGAAATTGTGGAAGTGCTGTTGAAGTACGGTGCAGATGTTAACGCGGCAGATGA<br>GGAGGGTgcCACGCCGCTGCATCTGGCAGCGAAAgcCGGTCACCTCGAAATTGTGGAAGTGCTGTTGAAGAATG<br>GTGCAGATGTGAATGCTCAGGATAAGTTTGGCAAAACCGCGTTTGATATCTCCATTGATAATGGCAACGAAGAT<br>TTAGCGGAAATCCTGCAGAAACTG

DARPin K28	GATCTGGGAAAAAAACTGCTGGAAGCCGCGCGTGCCGGGCAGGACGATGAGGTCCGTATTCTTATGGCGAACGG<br>TGCAGATGTTAACGCGTTCGATCACCACGGTTGGACGCCGCTGCATCTGGCAGCGCAACAAGGTCACCTCGAAA<br>TTGTGGAAGTGCTGTTGAAGTATGGTGCAGATGTTAACGCGGATGATCTGTTCGGTTACACGCCGCTGCATCTG<br>GCAGCGTGGAAAGGTCACCTCGAAATTGTGGAAGTGCTGTTGAAGTATGGTGCAGATGTTAACGCGATGGATCA<br>CCACGGTCACACGCCGCTGCATCTGGCAGCGCAAATGGGTCACCTCGAAATTGTGGAAGTGCTGTTGAAGTATG<br>GTGCAGATGTGAATGCTCAGGATAAGTTTGGCAAAACCGCGTTTGATATCTCCATTGATAATGGCAACGAAGAT<br>TTAGCGGAAATCCTGCAGAAACTG
DARPin K55	GATCTGGGAAAAAAACTGCTGGAAGCCGCGCGTGCCGGGCAGGACGATGAGGTCCGTATTCTTATGGCGAACGG<br>TGCAGATGTTAACGCGAACGATAGCGCAGGTCACACGCCGCTGCATCTGGCAGCGAAACGCGGTCACCTCGAAA<br>TTGTGGAAGTGCTGTTGAAGCATGGTGCAGATGTTAACGCGATGGATAACACGGGTTTCACGCCGCTGCATCTG<br>GCAGCGCTGCGCGGTCACCTCGAAATTGTGGAAGTGCTGTTGAAGAACGGTGCAGATGTTAACGCGCAAGATCG<br>CACGGGTCGCACGCCGCTGCATCTGGCAGCGAAACTGGGTCACCTCGAAATTGTGGAAGTGCTGTTGAAGAACG<br>GTGCAGATGTGAATGCTCAGGATAAGTTTGGCAAAACCGCGTTTGATATCTCCATTGATAATGGCAACGAAGAT<br>TTAGCGGAAATCCTGCAGAAACTG
